# The Influence of Physical Illumination on Lightness Perception in Simultaneous Contrast Displays

**DOI:** 10.1177/2041669518787212

**Published:** 2018-07-19

**Authors:** Daniele Zavagno, Olga Daneyko, Zili Liu

**Affiliations:** Department of Psychology, University of Milano-Bicocca, Italy; Department of Psychology, Sociology and Politics, Sheffield Hallam University, UK; Department of Psychology, University of California, Los Angeles, USA

**Keywords:** Gelb lighting, illumination, light, lightness constancy, lightness matching, lightness/brightness, simultaneous lightness contrast

## Abstract

Three experiments investigated the role of physical illumination on lightness perception in simultaneous lightness contrast (SLC). Four configurations were employed: the classic textbook version of the illusion and three configurations that produced either enhanced or reduced SLC. Experiment 1 tested the effect of ambient illumination on lightness perception. It simulated very dark environmental conditions that nevertheless still allowed perception of different shades of gray. Experiment 2 tested the effect of the intensity of Gelb lighting on lightness perception. Experiment 3 presented two conditions that integrated illumination conditions from Experiments 1 and 2. Our results demonstrated an illumination effect on both lightness matching and perceived SLC contrast: As the intensity of illumination increased, the target on the black background appeared lighter, while the target on the white background was little affected. We hypothesize the existence of two illumination ranges that affect lightness perception differently: *low* and *normal*. In the low range, the SLC contrast was reduced and targets appeared darker. In the normal range, the SLC contrast and lightness matchings for each background were little changed across illumination intensities.

## Introduction

We studied the effect of illumination on lightness perception in simultaneous lightness contrast (SLC) displays. With respect to the interaction between lightness and illumination, perceptual theories can be roughly grouped into two categories: those that consider lightness and illumination as connected processes (e.g., Agostini & Galmonte, 2002; Bergström, 1994; Blakeslee & McCourt, 2012; Gilchrist, 1979; Koffka, 1935; Logvinenko, Adelson, Ross, & Somers, 2005; Schirillo & Shevell, 2002; von Helmholtz, 1866/1962) and those that consider lightness processing as independent from illumination conditions (e.g., Bressan, 2006; Gilchrist et al., 1999; Rudd & Zemach, 2007; Todorović, 2006; Wallach, 1948). For a review on this topic, see Kingdom (2011). Here, we do not focus on the different theoretical stances about the relationship between lightness and perceived illumination; rather, we address the effect that the intensity of physical illumination has on lightness perception, separately from other factors in the perception of illumination (Zavagno, Daneyko, & Sakurai, 2011).

It is well established that lightness perception is greatly influenced by field factors—including local and global luminance ratios, and perceptual grouping—rather than by the luminance of an achromatic surface itself (Gilchrist, 1994, 2006). In general, this is desirable because luminance (i.e., the amount of light reflected away from a surface) is a variable source of visual information, whereas it is reasonable to assume that the goal of the visual system is to generate a model of the world in which an object’s structural features stay more or less constant despite changes over time in the retinal image (Zavagno, Daneyko, & Actis-Grosso, 2015). This is the phenomenon that goes by the name *perceptual constancy*.

However, one needs only to consider the extent of lightness/brightness illusions to appreciate that lightness constancy is a rather complex issue. Even if one wanted to dismiss lightness illusions as laboratory artefacts that have little to do with our experience of grays in the real world (Gibson, 1979), recent research suggests that lightness constancy in natural scenes is also quite poor, if not altogether bad (Baddeley & Attewell, 2009; Baddeley, Attewell, & Patel, 2010). This may be why much has been focused in the past four decades on studying lightness constancy failures, which are considered to be the key to understanding lightness perception (Gilchrist et al., 1999). Based upon Gilchrist et al.’s (1999) classification into Type 1 and Type 2 constancy, and upon Ross and Pessoa’s (2000) classification of lightness constancy as illumination-independent or as background-independent constancy, such failures have been classified into two types: (a) induced by illumination and (b) induced by the pattern of surface reflectance/luminance surrounding lightness targets. Both types are related to field factors that either introduce additional or modify existing visual information, thus affecting the luminance pattern surrounding the target. These field factors can give rise to different or even contradictory percepts for physically identical target surfaces. As our research focused on the influence of physical illumination on the perception of achromatic surface color, we employed experimental setups that envisage both types of failures by combining different types of SLC displays—capable of generating different perceptual effects on targets that are physically identical—with two types of illumination that can both be found in nature: ambient and direct.

In the literature, the most relevant article that deals with lightness and illumination is by Jameson and Hurvich (1961). These authors addressed “brightness constancy,” as was custom at the time. Nevertheless, they were actually speaking about achromatic surface color; hence, the appropriate term nowadays is ‘lightness constancy’. In fact, given the conceptualization of brightness in terms of perceived luminous energy, the term *brightness constancy* is a chimera from a theoretical point of view, as brightness is most often tightly correlated with luminance (Zavagno et al., 2011).^1^ Jameson and Hurvich reported in their study a slight increase in lightness matchings as the illumination over the targets was increased, except for two of their targets, a middle and a dark gray target. In the first case, no significant effect was found; in the second case, a darkening effect was found. Such findings suggest a nonlinear effect of illumination on lightness perception, a conclusion that contradicted the ‘luminance ratio rule’ advanced by Wallach (1948). According to this rule, the lightness of a target would appear constant if its luminance ratio to the background remained constant, which holds when illumination intensity changes.

Since the publication of Jameson and Hurvich (1961), at least four failures to replicate their findings have been reported (Flock & Noguchi, 1970; Haimson, 1974; Jacobsen & Gilchrist, 1988; Noguchi & Masuda, 1971; for a detailed account on the matter, see Gilchrist, 2006; for another prospective on the failures, see Taya, 1990). Moreover, Arend and Spehar (1993), in an experiment aimed at studying the effects of illumination on lightness and brightness, found that lightness matching performed by their observers was illuminance independent, even when local luminance contrasts at a target’s edge were not kept constant.

The experiments we describe here are not an attempt to replicate once more the work of Jameson and Hurvich (1961) or to replicate the work of Arend and Spehar (1993). Nevertheless, our study does bare some similarities to both studies, in the sense that we also wanted to test the role of physical illumination (i.e., illuminance) on surface lightness by modulating the illumination intensity on lightness displays. Apart from this, however, our study differs from the Jameson and Hurvich and the Arend and Spehar studies in the following important aspects: (a) the type of displays employed—paper versus surface projections in Jameson and Hurvich, and paper versus digital targets in Arend and Spehar; (b) how displays were illuminated—in Jameson and Hurvich, illumination was projected on the configuration; in Arend and Spehar, it was digitally simulated; in our case, we illuminated configurations either by light reflected from the walls or by pointing light sources directly on them (Gelb lighting, see Figure 2); (c) how illumination was modulated—luminance ranges for targets in the Jameson and Hurvich experiment was approximately 4:1 to 5:1; in the Arend and Spehar experiments, it was 19:1; in our experiments, the luminance range was overall 1500:1; and (d) how lightness effects were measured—both Jameson and Hurvich and Arend and Spehar employed a matching method by luminance adjustments, while we employed a matching method with a Munsell Neutral Value scale.

**Figure 1.**
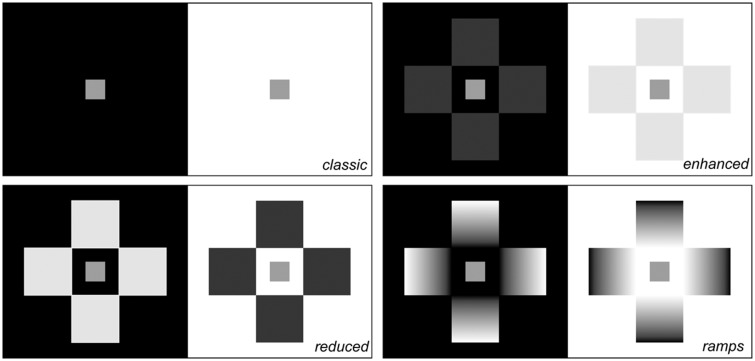
Configurations employed in all three experiments. While the classic SLC configuration is known to most psychology students, configurations dubbed as *reduced*, *enhanced*, and *ramps* are new and generate contrast levels different from the textbook version of the classic SLC illusion (Daneyko & Zavagno, 2008; Zavagno & Daneyko, 2012).

To summarize, in Experiment 1, we modulated the luminance of the configurations depicted in Figure 1 by modulating the amount of illumination in the laboratory; in Experiment 2, we modulated the luminance of the same configurations by modulating the intensity of Gelb lighting on those configurations.

**Figure 2.**
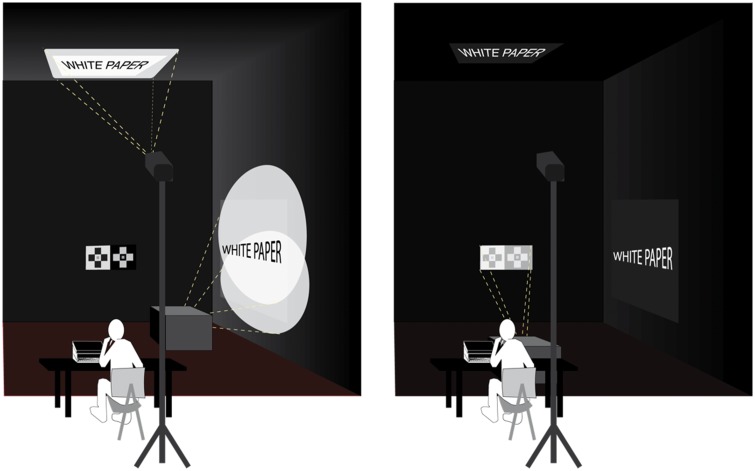
Schematic illustration of the lighting setups for Experiments 1 (left panel) and 2 (right panel). In Experiment 1, light configurations were illuminated by the light reflected by the walls and the ceiling, a portion of which was covered with white sheets of paper to increase ambient illumination; in Experiment 2, configurations were directly illuminated by a theatrical lamp hidden from sight.

## Experiment 1: Ambient Illumination

The purpose of Experiment 1 was to discover what would happen to lightness perception when the luminance of the stimulus pattern (in our case SLC configurations) was modified by increased or decreased intensity of ambient illumination. We dubbed this experiment “ambient illumination” because configurations were illuminated by secondary sources of illumination, that is, by the light reflected away by the walls in the lab. Because the laboratory was painted matte black, to increase the intensity of illumination on the configurations, we added additional sources of light, and we attached white sheets of paper on portions of walls that sided the display (approximately 1.5 m^2^ on each sidewall) and on a portion of the ceiling above the display (see Figure 2, left panel). We then manipulated target illumination by repositioning the added sources of room illumination to obtain three different intensities of target illumination. The additional sources of illumination were positioned on the floor, hidden from sight inside boxes, and directed at the portions of the sidewalls covered in white paper. This arrangement allowed us to manipulate illumination at will so as to illuminate the configurations homogenously.

### Participants

The total number of participants was 45: undergraduate, postgraduate, and PhD students from the University of Milano-Bicocca (26 female, mean age = 24.8, *SD* = 6.2). They were randomly assigned to one of the three groups. Each group was assigned to one condition of illumination (between-subjects factor) and saw all four configurations (within-subjects factor), one at a time in random order. While participants might have been exposed to the classic SLC configuration, which can be easily found in first-year undergraduate textbooks, they had no previous exposure to the three modified SLC configurations. All participants had normal or corrected-to-normal vision. All experiments in this study were conducted in accordance with the guidelines outlined in the Declaration of Helsinki.

### Materials

The SLC configurations employed were those depicted in Figure 1. The black background and the surrounding squares were created with a high-definition inkjet printer (Epson Stylus Photo R2400; the paper was 167 g/m^2^ Epson matt white paper). The background size of each target was 140 × 140 mm; the size of each target was 20 × 20 mm; in the modified SLC configurations, each square was 40 × 40 mm (Figure 1). The targets were cut out from actual 5.0 Munsell neutral value paper. The stimulus configurations were fixed to the end of a rod that was 50 cm long and attached to the back wall, thus appearing suspended in midair. In addition to the room’s neon lighting, a set of lamps were added: two mini LED theatrical spotlights (SPOTLIGHT Mini PR model ME, 18000 lm, 6800 K) and two LED table lamps pointed against the sidewalls, and another theatrical spotlight (“Acclaim” ZOOM PROFILE 18-34 by SPOTLIGHT, with T27 650W halogen lamp, 15500 lm, 3000 K) positioned 1.5 m behind the participant and pointed toward the portion of the ceiling covered with white paper. Three room illumination intensities were thus determined by directing the lamps to different wall areas; the different levels of room illumination were dubbed *normal*, *low*, and *dark*. The luminance readings of the configurations, measured with a TOPCON Luminance Colorimeter BM-7 A, are reported in Table 1.

**Table 1.** Luminance Readings (cd/m^2^) for the Four Configurations Viewed Under the Three Room Illumination Conditions.

	Normal	Low	Dark
Target	13.50	3.50	0.40
Black background	1.93	0.56	0.05
White background	61.80	15.80	1.27
Light cross	46.00	11.51	1.00
Dark cross	9.17	2.20	0.25

**Figure 3.**
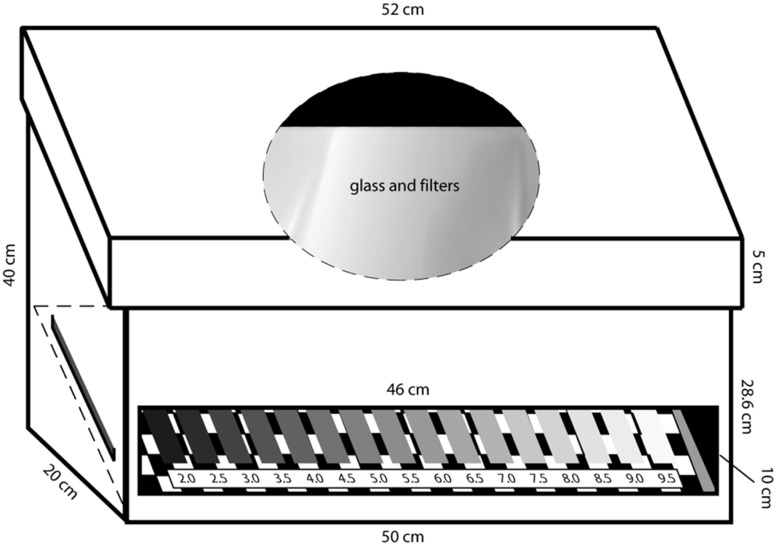
Schematic illustration of the matching box with the 16-step Munsell scale illuminated separately by LED light. The term *filters* refers to achromatic diffusion filters added to diffuse in a more even way illumination within the box (see Zavagno et al., 2011 for details).

A matching method was used with a 16-step lightness scale ranging from Munsell n.v. 2.0 to 9.5 (Zavagno, Daneyko, & Agostini, 2011). Such a scale was seen against a printed black–white checkerboard background and inserted inside a viewing box (Figure 3) with its own constant illumination that had no effect on the laboratory’s illumination. Within the scale, Step 5.0 had luminance 13 cd/m^2^; and Steps 2.0 and 9.5 were 1.7 and 51 cd/m^2^, respectively.

### Procedure

Participants entered the lab that was already set with the proper illumination level. After they were seated, their personal data were recorded. All participants first took part in an experiment on haptic perception, which allowed them to adapt to the laboratory’s illumination. That experiment lasted about 25 min, after which an SLC configuration, randomly chosen from the four, was revealed to the participant. The participant was instructed about the meaning of ‘target’ and ‘lightness’ and was shown the matching scale placed 25 cm to their left. It was explained that the task was to find the closest match possible for each target in each configuration, as if the match on the scale and the target was cut out from the same paper. If the participant had no questions, the first trial started, after which the participant was required to lower her or his head while one of the two experimenters changed the stimulus. The display was positioned 270 cm away from the participant. The experiment lasted about 10 min.

### Results and Discussion

**Figure 4.**
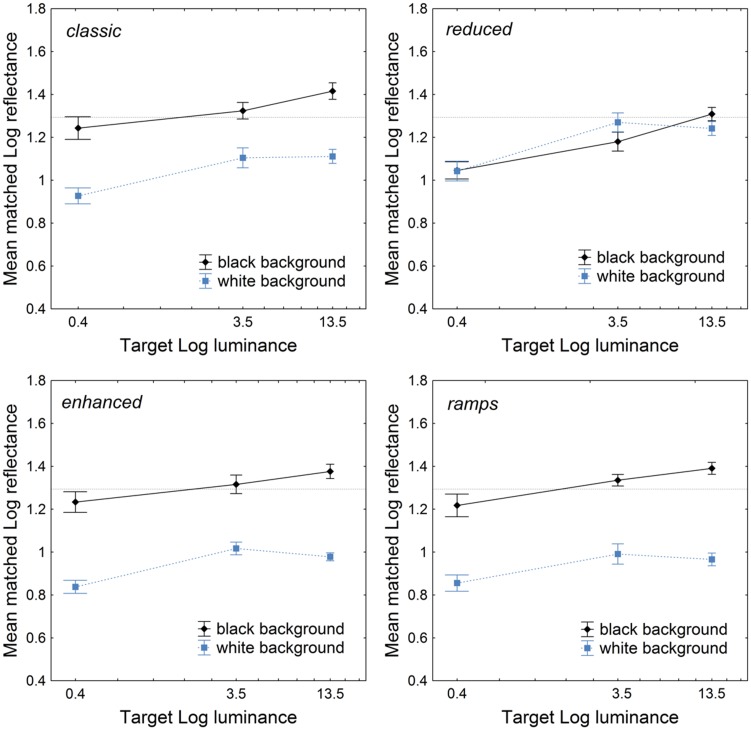
Results for Experiment 1. The *x* axis reports target luminance on a log scale. The horizontal dashed line indicates targets’ actual log reflectance; vertical bars denote standard errors. Among the three modified SLC configurations, only the *reduced* gave rise to matching results that differed from the classic display.

Figure 4 shows results in terms of the target mean matched log reflectance for each SLC configuration that was viewed under each of the three illumination conditions (the targets’ actual log reflectance was 1.29). Analyses of variance (ANOVAs) for repeated measures were carried out on the matched log reflectance data separately for each configuration (classic, reduced, enhanced, and ramps), with *Background* (black or white) as a within-subjects factor and *Illumination* as a between-subjects factor. With the exception of the configuration *reduced* (*p* = .8), the factor *Background* produced significant effects on target matches, as expected—*classic*: *F*(1, 42) = 71.77, *p* < 10^−4^, η_p_^2 ^= 0.63; *enhanced*: *F*(1, 42) = 181.88, *p* < 10^−4^, η_p_^2 ^= 0.81; and *ramps*: *F*(1, 42) = 190.43, *p* < 10^−4^, η_p_^2 ^= 0.81. *Illumination* produced significant effects in all four configurations, though the effects were stronger with the configuration reduced (as evidenced also by the reported effect size η_p_^2^)—*classic*: *F*(2, 42) = 9.41, *p* < .001, η_p_^2 ^= 0.30; *reduced*: *F*(2, 42) = 16.27, *p* < 10^−4^, η_p_^2 ^= 0.43; *enhanced*: *F*(2, 42) = 8.87, *p* < .001, η_p_^2 ^= 0.29; and *ramps*: *F*(2, 42) = 6.60, *p* < .005, η_p_^2 ^= 0.23. None of the interactions *Background* × *Illumination* were significant.

As one can notice, with the exception of the configuration *reduced*, the other two modified SLC configurations gave rise to lightness matches that were in line with the configuration *classic.* From this experiment, we concluded that, with the exception of the configuration *reduced*, additional luminance information did not affect lightness matches, while the intensity of ambient illumination did indeed affect lightness perception in all configurations. Did it affect the magnitude of the contrast illusion as well? For each participant, the magnitude of the illusion was calculated as the difference between the matched Munsell value of the target on the black background and that on the white background; negative differences denote a reverse contrast effect (Economou, Zdravkovic, & Gilchrist, 2007). An ANOVA for repeated measures was conducted on the contrast magnitude data, with *Configuration* as the within- and *Illumination* as between-subjects factors: *Configuration* produced a significant main effect, *F*(3, 126) = 49.53, *p* < 10^−4^, η_p_^2 ^= 0.54, while neither the *Illumination* nor the interaction *Configuration* × *Illumination* yielded significant effects on contrast magnitudes (*p* > .08).

## Experiment 2: The Gelb Lighting

In Experiment 1, we wanted to test what happened in a setup where illumination may vary, but the display is illuminated by secondary sources of illumination. In nature, it may well be that targets are directly illuminated by, say, a primary source of illumination, such as sunlight, or often by a combination of primary and secondary sources of illumination. To simulate a condition when a beam of light is directly focused on a surface, we conducted an experiment that employed Gelb illumination. Differently from how Gelb illumination is usually employed, which is to illuminate exclusively a target or a set of targets (Cataliotti & Gilchrist, 1995; Gelb, 1929), our Gelb lighting illuminated the entire SLC configurations (targets and their immediate surroundings), and nothing else. We employed the Gelb lighting this way to keep luminance ratios constant within a configuration viewed under different intensities of the Gelb lighting (Wallach, 1948). To modulate the intensity of the illumination, we used neutral density filters applied directly to the lamp illuminating the configurations. The Gelb illumination is not, of course, a natural setup, as it takes place in a room where a spotlight that is directed on the configuration constitutes the only means of illumination. This makes it a useful tool to study pure luminance effects on lightness in real-world settings. The laboratory setup was otherwise the same as in Experiment 1, with white paper attached to the walls siding the displays and white panels attached to a portion of the ceiling above the display. As the intensity of the Gelb lightning was increased, the room brightness could increase also, when compared with what would have occurred in a plain black laboratory. To sample how the intensity of Gelb lighting had affected the brightness of our laboratory, we measured the luminance of the same area (approximately 7 cm in diameter; distance from target = 135 cm) on the sidewall in the laboratory that was covered with white paper for the different Gelb lighting intensities (see bottom row in Table 2).

**Table 2.** Luminance Readings (cd/m^2^) for the Four Configurations Viewed Under the Six Gelb Illumination Levels.

	0 f/stop	1 f/stop	2 f/stop	3 f/stop	6 f/stop	9 f/stop
Target	269	127	61.7	28.7	3.2	0.53
Black background	34.5	15.42	8.55	3.72	0.42	0.07
White background	1112	522	259	119	13.51	1.7
Dark cross	162	77.37	37.47	17.77	2.21	0.35
Light cross	845	401	193	92.17	10.61	1.5
Sidewall white paper	2.24	1.1	0.52	0.25	0.032	0.004

*Note*. As f/stop increases, the luminance readings decrease. The bottom row of the table reports luminance readings for the same spot of white paper on one sidewall of the laboratory.

### Participants

Participants were 66 undergraduate, graduate, and postgraduate students and researchers (35 female, mean age = 25.4, *SD* = 8.11) from the University of Milano-Bicocca who did not take part in any of the previous studies. Participants were randomly assigned to one of six groups of Gelb illumination intensities. All participants had normal or corrected-to-normal vision.

### Materials and Procedure

The viewing distance, SLC configurations, and Munsell matching scale were the same as in Experiment 1. The displays were illuminated by an LED stage lamp (SPOTLIGHT mini PR model ME) positioned on the floor and hidden from view. The beam of light was shaped to illuminate the entire SLC configuration and to reduce light diffusion. The presence of a source of light illuminating the stimuli was evident, as this was the only source of illumination inside the laboratory. The position of the lamp was however such that the light not falling on a configuration was out of sight from the position where participants were seated. The intensity of the beam was modulated by photographic high-temperature-resistant neutral density gels (Norman, Bartlett, IL, USA). We cannot exclude the possibility that, at the highest level of the Gelb illumination, the white backgrounds could have appeared super white or even glowing. However, none of the participants complained about any discomforting glare (Facchin, Zavagno, & Daini, 2017), nor did they report any self-luminous targets. Neutral density filters were made in such a way that they reduced illumination nearly evenly across the visible spectrum, and their light reduction effect is described in terms of f/stops, a photography notation (for a simple and elegant explanation of basic concepts, see http://www.outdoorphotoacademy.com/f-stops-made-simple/). By combining these filters, we obtained six Gelb illumination intensities that we dubbed as follows: 0 f/stop (unfiltered Gelb illumination), 1, 2, 3, 6, and 9 f/stop. The beam of light became weaker as the f/stop increased. Table 2 shows the main luminance values of the displays for each Gelb illumination condition. The experimental procedure was identical to that described in Experiment 1.

### Results and Discussion

**Figure 5.**
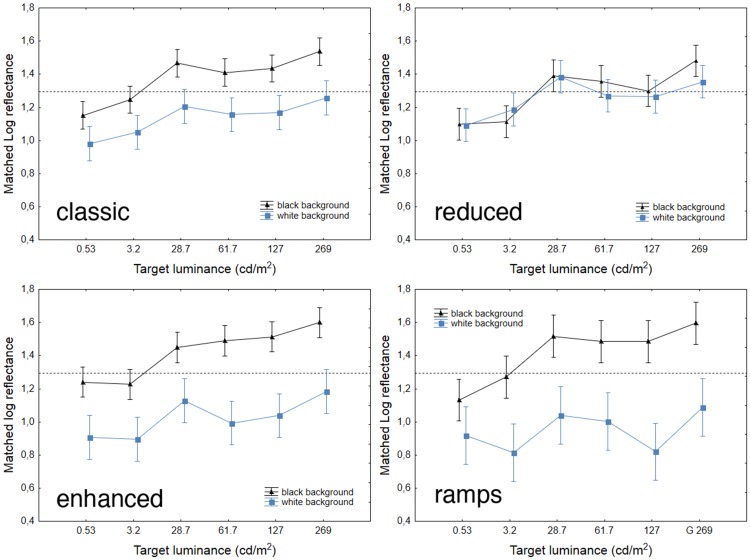
Results for Experiment 2. The *x* axis shows target luminance in log scale. The horizontal dashed lines indicate targets’ actual log reflectance. The vertical bars denote standard errors of the means, as the rest of the figures.

Figure 5 displays the results for the six intensities of the Gelb lightning in log reflectance matchings for each configuration. ANOVAs for repeated measures were conducted similarly as in Experiment 1, with *Background* as the within- and *Illumination* as the between-subjects factors. As expected with SLC configurations, with the exception of the configuration *reduced* (*p* = .2), *Background* gave rise to a significant main effect on target matchings in the other three configurations—*classic*: *F*(1, 60) = 114.47, *p* < 10^−4^, η_p_^2 ^= 0.65; *enhanced*: *F*(1, 60) = 136.59, *p* < 10^−4^, η_p_^2 ^= 0.69; and *ramps*: *F*(1, 60) = 104.27, *p* < 10^−4^, η_p_^2 ^= 0.63. As in Experiment 1, *Illumination* gave rise to a significant main effect on matchings for all four configurations—*classic*: *F*(5, 60) = 10.78, *p* < 10^−4^, η_p_^2 ^= 0.47; *reduced*: *F*(5, 60) = 12.02, *p* < 10^−4^, η_p_^2 ^= 0.5; *enhanced*: *F*(5, 60) = 11.20, *p* < 10^−4^, η_p_^2 ^= 0.48; *ramps*: *F*(5, 60) = 6.41, *p* < 10^−4^, η_p_^2 ^= 0.34. None of the interactions *Background* × *Illumination* effects were significant (*p* > .1).

Results with filters 6 f/stop (3.2 cd/m^2^) and 9 f/stop (0.53 cd/m^2^) were similar to the results in Experiment 1 in the low (3.5 cd/m^2^) and dark (0.4 cd/m^2^) illumination conditions (see Figure 3), with the target on the black background appearing either darker or virtually equivalent to its actual Munsell value. From 0 to 3 f/stop, the SLC illusion was what one would expect, appearing roughly constant from one illumination intensity to the other. This finding suggests an effect of illumination intensity on target lightness acting within two ranges, which we dubbed as *low* and *normal*. Within the low range, targets on the black background were seen as darker than when they were viewed in the normal range. For targets on the white background, the effect of illumination was less pronounced in that the targets still appeared darker in the *low* than in the *normal* range, but this difference seemed smaller than in the case of the black background.

As in Experiment 1, we calculated the magnitude of SLC for each configuration and for each participant. An ANOVA for repeated measures was conducted on such data, revealing significant main effects of both the within-subjects factor of *Configuration* and the between-subjects factor of *Illumination*, *F*(3, 180) = 65.09, *p* < 10^−4^, η_p_^2 ^= 0.52; *F*(5, 60) = 2.86, *p* < .05, η_p_^2 ^= 0.19, respectively, while their interaction was not significant (*p* = .3).

In sum, lightness matchings of the SLC illusion support the hypothesis of two distinct illumination ranges that affect lightness perception in different ways. But how much did the results of Experiment 2 depend on the impact of the Gelb lighting on the intensity of the laboratory’s illumination?

To our knowledge, we are the first to have measured ambient luminance variations in relation to the intensity of Gelb lighting in settings in which that lighting is the only source of ambient illumination, which has always been implicitly assumed to have no influence on ambient illumination. However, even if the Gelb lighting was perfect—that is, illuminating perfectly and uniformly only the configuration under study in real-life settings—the light reflected from the configuration would still affect ambient illumination somewhat. For instance, Agostini and Bruno (1996), in describing the illumination of the room with white walls where they ran their Gelb lighting condition, reported that

A rectangular beam of light from an adjustable halogen lamp was cast on the wall so that its illumination edges coincided exactly with the outer border of the display. *This condition was run in a large room dimly illuminated by indirect light that also originated from the halogen lamp*. (p. 252; the italics are ours)

In our study, interreflections were less pronounced because a relatively small portion of the laboratory’s black walls and ceiling were covered in white. Hence, though ambient illumination did vary in intensity somewhat, these variations were likely inconsequential with regard to the effects of Gelb lighting in our experiment because of the low luminance readings (see Table 2), which can be considered as a very good approximation of perfect Gelb lighting.

**Figure 6.**
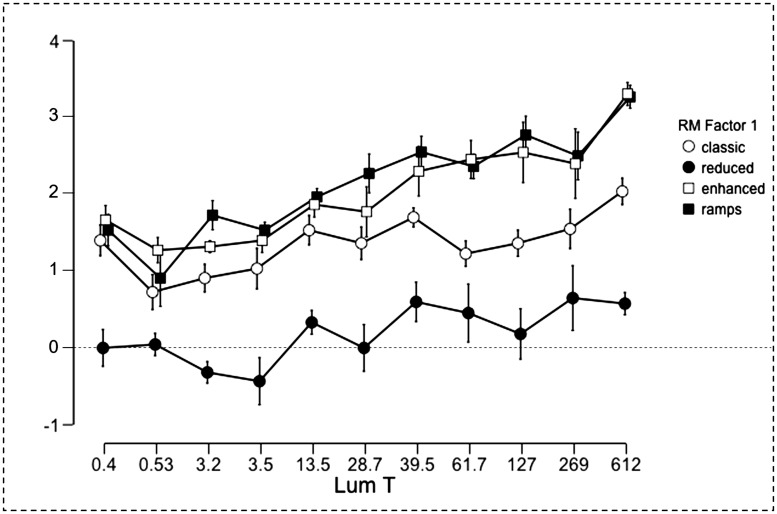
Mean SLC magnitude for all illumination conditions, expressed as Munsell units (*y* axis) as a difference between the target on the black background and the target on the white background (negative values denote inverse contrast). The *x* axis reports target luminance.

There is, however, a second issue relevant to the Gelb lighting that needs to be addressed. Namely, is it a special case of illumination, and does it enhance the magnitude of lightness illusions (Gilchrist, 2016)? To our knowledge, there are no studies dedicated to the effect of the intensity of the Gelb illumination on the magnitude of lightness illusions. It is reasonable to assume that the Gelb lighting has an “isolation” effect on stimuli. Agostini and Bruno (1996) showed that the magnitude of the SLC illusion was greater when the spotlight illuminating the display was shaped so as to coincide with the configuration itself. As the area of illumination increased, the magnitude of the illusion decreased. These findings strongly support the hypothesis that Gelb lighting exerts an isolation effect (Gilchrist, 2016). According to the anchoring theory (Gilchrist et al., 1999), an SLC configuration illuminated by Gelb lighting in a dim room would undergo lightness computations partially or totally separated from the surrounding environment not directly illuminated by the Gelb lighting. In the case of classic SLC, this ought to translate into stronger differences between targets depending on the degree of isolation of the SLC configuration from the rest of the laboratory because of the Gelb lighting. However, based on such reasoning, Gelb lightings of lower intensity should induce even a stronger lightness illusion, as light scattering (and therefore ambient brightness) is greatly reduced, and consequently, the illuminated configurations are virtually completely isolated from the rest of the environment. Nevertheless, the contrast effects in our Experiment 2 were weaker at the lowest Gelb lighting intensities (Figure 6). Moreover, if the Gelb lighting produced an isolation effect irrespective of its intensity, the lightness of the SLC targets should have been little affected by the variations in the intensity of the Gelb lighting. In other words, both target lightness and the magnitude of the contrast illusion should not have been affected by the changes in intensity of the Gelb lighting. Instead, the magnitude of the contrast illusion increased approximately one Munsell step from the *low* to the *normal* illumination range for the configuration *classic*. This finding suggests that the effect of the Gelb lighting on lightness targets was affected by its intensity. Nevertheless, the isolation hypothesis is not necessarily confuted by the findings of Experiment 2, given that at the lowest Gelb intensities we might have entered or bordered the scotopic luminous efficiency range, an illumination condition in which only rods are active (Schubert, 2006). According to Rudd and Rieke (2016), under such viewing conditions, the Weber’s law does not hold, and this may account for the general darkening of the targets. This point is further considered in the General Discussion section.

## Experiment 3: Extensions of Experiments 1 and 2

The purpose of Experiment 3 was to address an issue still open in the comparison between Experiments 1 and 2 and to test what would happen to lightness perception in SLC configurations when the intensity of the Gelb lighting was further increased.

The issue is as follows: The luminance intensity of the targets in the *normal* illumination condition in Experiment 1 (13.5 cd/m^2^) was less than half of the intensity of the targets that were viewed under the *normal* range of illumination in Experiment 2 (for instance, the targets’ luminance viewed under 3 f/stop was 28.7 cd/m^2^). We therefore decided to set up at least one condition of ambient illumination where the luminance of targets would be comparable with the luminance of targets in Experiment 2 in conditions 2 and 3 f/stop (*extension of Experiment 1*).

Based on the results from Experiments 1 and 2, we hypothesized the existence of at least two illumination ranges, within each of which lightness and the magnitude of the contrast effects were only mildly affected by the intensity of illumination, when luminance ratios within the configuration remain constant. We wanted to explore what would happen if we further increased the intensity of the Gelb illumination (*extension of Experiment 2*).

### Participants

Participants were 33 undergraduate and graduate students (mean age = 24.6, *SD* = 4.9) from the University of Milano-Bicocca who did not participate in any of the previous experiments. Twenty (14 female) participated in the extension of Experiment 1, and 13 (6 female) in the extension of Experiment 2. All participants had normal or corrected-to-normal vision.

### Materials and Procedure

The SLC displays and matching scale were the same as before. For the extension of Experiment 1, by rearranging the lamps used in Experiment 1, we generated a condition in which the target luminance was 39.5 cd/m^2^, which was between those targets viewed under 2 and 3 f/stop Gelb illumination in Experiment 2 (61.7 and 28.7 cd/m^2^, respectively). For the extension of Experiment 2, two identical theatrical LED spot lights were employed (SPOTLIGHT Mini PR model ME). In addition, a relatively strong stage light, the same as in Experiment 1, positioned behind the observer, was pointed toward the ceiling. This light was added to avoid a dazzling effect that might have occurred if the double Gelb lighting was used as the only means of ambient illumination. This new condition, therefore, added a new interesting situation: the Gelb lighting within an illuminated room, which combines and extends Experiments 1 and 2. Table 3 shows the luminance readings for the main features in the SLC configurations.

**Table 3.** Photometric Readings in cd/m^2^ in Experiment 3.

	Extension of Experiment 1	Extension of Experiment 2
Target	39.5	612
Black background	5.6	98.27
White background	180	2812
Light cross	133.6	2166
Dark cross	27	456

The procedure was the same as before, including the adaptation time to lab illumination by means of a haptic experiment.

### Results and Discussion

The matching results of the extension of Experiment 1 (more intense ambient illumination) were in line with the results from Experiment 2 within illumination conditions that we defined as a ‘normal illumination range’ (1, 2, and 3 f/stop). Unpaired *t* tests were carried out with log reflectance data to compare the matchings between targets 39.5 cd/m^2^ (black and white backgrounds) and targets 28.7 (3 f/stop), 61.7 (2 f/stop), 127 (1 f/stop), 269 (0 f/stop), and also the “double Gelb” condition (extension of Experiment 2, target 612 cd/m^2^). Table 4 shows the mean Munsell matches and the *t* test *p* values. With regard to the black backgrounds, the matchings for Target 39.5 cd/m^2^ differed significantly only from the matchings for Target 612 cd/m^2^, with exception of configuration *reduced*. With regard to the white backgrounds, the mean of Target 39.5 cd/m^2^ differed significantly only from the mean for Target 612 cd/m^2^ in the configuration *classic*.

**Table 4.** Extension to Experiment 1: Mean Munsell Matches and *p* Values From Unpaired Two-Tailed *t* Tests With Log Reflectance Data.

	*E3*, *T39.5*	*E2*, *T28.7*	*E2*, *T61.7*	*E2*, *T127*	*E2*, *T269*	*E3*, *T612*
Black background						
Classic	Muns = 5.8	Muns = 5.7; *p* > .5	Muns = 5.6; *p* > .3	Muns = 5.7; *p* > .7	Muns = 6.4; *p* = .051	Muns = 6.7; *p* < .005
Reduced	Muns = 5.7	Muns = 5.5; *p* > .6	Muns = 5.3; *p* > .2	Muns = 5; *p* = .054	Muns = 6; *p* > .1	Muns = 5.9; *p* > .3
Enhanced	Muns = 6.3	Muns = 6; *p* > .1	Muns = 6.1; *p* > .2	Muns = 6.2; *p* > .6	Muns = 6.8; *p* > .2	Muns = 7.2; *p* < .01
Ramps	Muns = 6.3	Muns = 6.2; *p* > .8	Muns = 6; *p* > .4	Muns = 6; *p* > .4	Muns = 6.7; *p* > .1	Muns = 7; *p* < .05
White background						
Classic	Muns = 4.1	Muns = 4.5; *p* < .05	Muns = 4.4; *p* > .4	Muns = 4.4; *p* > .1	Muns = 4.9; *p* = .059	Muns = 4.6; *p* < .05
Reduced	Muns = 5.1	Muns = 5.5; *p* > .2	Muns = 4.9; *p* > .5	Muns = 4.9; *p* > .5	Muns = 5.3; *p* > .4	Muns = 5.3; *p* > .3
Enhanced	Muns = 4	Muns = 4.2; *p* > .4	Muns = 3.6; *p* > .4	Muns = 3.6; *p* > .8	Muns = 4.5; *p* > .1	Muns = 3.9; *p* > .9
Ramps	Muns = 3.7	Muns = 4; *p* > .7	Muns = 3.7; *p* > .8	Muns = 3.3; *p* = .07	Muns = 4.2; *p* > .5	Muns = 3.8; *p* > .9

In sum, results from extension of Experiment 1 confirmed that if the intensity of ambient illumination was such that the target luminance fell within the range we dubbed as *normal*, then the lightness matchings for those targets were in line with the lightness matchings under the Gelb lighting that was also within the normal illumination range.

The matching results from the double Gelb lighting (extension of Experiment 2) showed a slight increment in the magnitude of the illusion (see Table 4 for matching results in Munsell units). Unpaired *t* tests were conducted, with log reflectance matching data for the targets on the black and on the white backgrounds, to compare matchings between targets 612, 127, and 269 cd/m^2^. With regard to the black background, the comparison between Targets 612 and 127 was significant for all configurations—classic: *t*(22) = −2.61, *p* = .016; reduced: *t*(22) = −2.57, *p* = .017; enhanced: *t*(22) = −2.53, *p* = .019; ramps: *t*(22) =−3.11, *p* < .005. The comparisons between Targets 612 and 269 cd/m^2^ and between Targets 269 and 127 cd/m^2^ were instead not significant in any configurations (*p* > .06). With regard to the white background, none of the comparisons were significant (*p* > .06).

In our quest to understanding what happens when the Gelb lighting is further increased, for each configuration, we conducted an ANOVA with the Magnitude data (calculated as stated for Experiment 1) from all experiments with *Illumination* as between-subjects factor. *Illumination* gave rise to significant effects only with configurations *enhanced* and *ramps*: *F*(10, 132) = 3.80, *p* < .001, η_p_^2 ^= 0.22; and *F*(10, 132) = 4.30, *p* < .001, η_p_^2 ^= 0.24, respectively. With regard to conditions *classic* and *reduced*, the main effect of *Illumination* was only marginally significant (*p* = .051 and *p* = .059, respectively). Figure 6 plots all the magnitude data for the four configurations. While the graph shows a general increment in contrast magnitude as target luminance increases, the ANOVAs and post hoc tests (Tukey and Bonferroni) tell a different story. The magnitude of the contrast effect in the double Gelb lighting condition (target luminance 612 cd/m^2^) was significantly different only from the magnitudes obtained in the darkest illumination conditions (from 0.4 to 3.5 cd/m^2^) and only for configurations *enhanced* and *ramps* (*p* < .01). Such results support a two-range hypothesis, but keep open the question of what happens to the SLC illusion when target luminance is even further increased.

## General Discussion

Experiment 1 was designed to test the effect of ambient illumination on surface lightness perception. The classic and modified SLC configurations were chosen as stimuli to underscore the effects of the intensity of illumination; however, under those illumination conditions, only lightness matches for the configuration *reduced* appeared significantly different from those for the classic SLC configuration. We found an effect of illumination on lightness but not on the magnitude of SLC. Specifically, illumination appeared to affect mostly the target on the black background, which appeared lighter in the highest illumination (Figure 4, target luminance 13.5 cd/m^2^). This finding is in line with the data that emerged from the *locus of error* paradigm developed by Gilchrist et al. (1999) and Economou et al. (2007), and with Rudd and Rieke’s (2016) findings for rod vision.

Experiment 2 employed the same stimuli as Experiment 1, which were viewed under the Gelb lighting. We found an effect of illumination on lightness and also on the magnitude of SLC in all configurations. Lightness evaluations and SLC magnitudes for targets viewed under the Gelb lighting 6 and 9 f/stop were not statistically distinguishable from targets in corresponding configurations viewed in dark and low room illuminations in Experiment 1.

The overall results from Experiments 1 and 2 (but not yet Experiment 3) suggest two hypotheses that require further investigations: (a) the Gelb lighting did not constitute a special condition per se, in the sense that it did not modulate lightness in a special way, any differently from ambient illumination, and (b) illumination intensities could be roughly grouped into two ranges in reference to the effects they had on lightness perception. We named such ranges as *low* and *normal*.

With regard to the second point, the low range showed a general darkening of SLC targets in all configurations. This finding implies that the Weber’s law—and therefore constancy—may not hold up under scotopic conditions (for a thorough discussion on the issue, see Rudd & Rieke, 2016). However, we cannot ensure that the spectral sensitivity of ambient illumination condition was within a scotopic luminous efficiency range in the illumination conditions that fall within the low range in Experiments 1 and 2, given that the methods for calculating lighting levels in the existing literature actually refer only to a “luminous quantity” that falls on a surface (Saunders, Jarvis, & Wathes, 2008). What this implies, but not acknowledged in the literature, is a correlation between surface brightness and the brightness of the visual field. This correlation has only predictive value because surface brightness is implicitly treated as a rough manifestation of the brightness of the visual field (assuming that the latter is homogeneous, which is a strong assumption). This however makes sense only for extremely simplified experimental setups aimed at measuring sensitivity functions and thresholds. With reference to our experiments, such assumption is nonapplicable, given that neither the size of the environment nor the articulation of the visual field is represented in the equations. This said, we might have approached scotopic vision only for the Gelb lighting condition at 9 f/stop (see Table 2), in which the luminance readings for the configuration were actually very low, and the luminance reading for the white paper attached to the sidewalls bordered the luminance conditions for scotopic vision (Schubert, 2006). Most of the other low-level illumination conditions that we grouped under the label *low range* might instead fall within the mesopic vision range, an intermediate condition when rods gradually become sensitive and cones are still active (Packer & Williams, 2003; Schubert, 2006; Zele & Cao, 2015).

The normal range, in comparison, comprised illumination levels within which lightness constancy held. These effects applied to our *classic*, *enhanced*, and *ramp* SLC displays; and also in part to the *reduced* SLC display, in which the illusion appeared either weak, or sometimes reversed. Finally, in the normal range, lightness matches and contrast magnitudes for the configurations *reduced*, *enhanced*, and *ramps* were significantly different from those for the classic configuration (see Figures 5 and 6).

Experiment 3 was designed to (a) integrate findings from Experiment 1 and 2 by using a level of ambient illumination comparable with the normal illumination range as defined by Experiment 2 (Experiment 3, Extension 1) and (b) test what happens to SLC when the intensity of the Gelb lighting is further increased (Experiment 3, Extension 2). The results from Extension 1 do not support the hypothesis that the Gelb illumination is special in increasing the illusion in SLC displays, because lightness matches were statistically indistinguishable within the *normal* range, regardless of the type of illumination (Gelb or ambient). The results from Extension 2 are, instead, somewhat inconclusive: On one hand, matching results for the target on the black background appear to be affected by the increased illumination, appearing lighter; on the other hand, the magnitude of the illusion is not statistically different from the other illumination conditions grouped within the *normal* range.

To summarize: In the low illumination range, the perceived lightness was compressed downward on the lightness scale: Eventually, when the environment gets too dark, one would not be able to make out subtle lightness differences, though still be able to perceive surfaces. One might be tempted to consider the low range as taking place within scotopic illumination conditions. This would be convenient, as Rudd and Rieke (2016) offered an account on lightness constancy failures in scotopic vision based on findings derived from experiments aimed at studying the properties of the brightness gain control function. However, we believe that their account might apply to the darkest Gelb illumination condition (9 f/stop). In fact, as we stated earlier, we cannot be sure that our other illumination conditions fell within the scotopic luminous efficiency range. Moreover, when it comes to SLC configurations, it is important to underscore that lightness constancy Type 2 (background effects) is never feasible at any level of illumination, except perhaps for displays similar to our configuration *reduced*. Having said that, what we think Rudd and Rieke’s analysis can certainly account for is the drop in perceived lightness in rod vision, when targets appear generally darker than they would otherwise appear in a normal range of illumination. More experiments are required to test what happens to lightness when the illumination of SLC configurations, in both ambient and Gelb lighting setups, drops even lower.

How should one relate the illumination effect on lightness perception that we report here to previous findings in which lightness was reported to be relatively immune to illumination changes? While the total number of such studies is small, those studies somewhat similar to ours seem to share a common feature: The experiments carried out were computer-based, with stimuli presented on CRT screens (e.g., Arend & Spehar, 1993; MacEvoy & Paradiso, 2001). This has two important implications. The first is that illumination modulations were simulated on the computer screen by modulating luminance values in such a way to keep unaltered local luminance ratios. As people who work with paper displays know, it is hard and time consuming to achieve constant local luminance ratios as illumination conditions are altered. Moreover, luminance alterations within the visual field will always come with some “noise” in the luminance readings of the surfaces involved in the experiment. This noise, however, may be relevant by making the visual scene appear more natural, that is, ecologically more valid, as the observer also participates within the environment under observation. Hence, what we consider to be noise may be a level of information that at the moment we fail to recognize and therefore to model (see, for instance, Zavagno and Caputo, 2005 for the role of “luminance noise” in luminosity perception). It is also possible that some of the noise may in fact be inherent to surface structure (that is, to its *skin* or *mesostructure* as it is now referred to), and how this might influence lightness is currently a new topic of research and discussion (Anderson & Kim, 2009; Motoyoshi, Nishida, Sharan, & Adelson, 2007; Schmid & Anderson, 2014). In computer-generated displays, the participant is ‘locked out’ of the scene. Hence, it is problematic to claim that observers were presented with scenes illuminated with different intensities if such intensities did not affect the observers’ environment, or if no direct question was made concerning the levels of illumination. The point we are making here does not concern the validity of computer-based studies on lightness and brightness: As everyone in the field knows, computer-based experiments are powerful tools for studying the visual system at work in highly controlled conditions (for a discussion on the matter, see Gilchrist, 2016). What we are saying is that such controlled conditions may also become a form of isolation from the world, and therefore, one might expect differences in results coming from how stimuli are presented, even when physical parameters between presentation modes (i.e., computer-based vs. so-called ecological) appear to match (see, for instance, Agostini & Bruno, 1996). The point is that even when the conditions of stimuli presentations in real-world setups are far from being ecological, as with the Gelb Lighting, participants are still part of the environment that contains the stimuli, be it dark or bright. When instead stimuli are presented on a computer screen, the participant is seeing stimuli that pertain to another environment, separated from their own. How this may affect lightness evaluations is an intriguing question.

While the first implication is at the moment still theoretical speculation, there is a second implication that is factual: CRT-generated patterns can display only a limited range of luminance values. In our experiments, the luminance ranged overall 56000:1 across conditions. This means that even if the CRT-based experiments succeeded in mimicking illumination, all medium to high luminance values would fall well within what would be displayed within the hypothesized range of illumination that we defined as *normal* in our study. Given the luminance range limitations within CRT-based experiments, target lightness for specific backgrounds in SLC displays should appear constant despite changes in simulated illumination.

On the other hand, the luminance range we employed is comparable, though only roughly, to that employed by Jacobsen and Gilchrist (1988). In trying and failing to replicate Jameson and Hurvich (1961), they used actual light to illuminate paper stimuli and found lightness perception to be quite constant across a range of four levels of illumination intensity. However, we ought to consider only the binocular condition in their experiment with the Munsell matching technique, roughly comparable with ours, in which only three of the four illumination intensities were employed, the darkest being left out. The difficulty of pitting our findings against theirs lies in two relevant points. First, their stimuli replicate, with greater ecological validity, the configuration employed by Jameson and Hurvich where targets were adjacent to each other forming a cross pattern. Second, stimuli were observed by looking through an aperture; hence, manipulations of illumination intensity affected only the stimulus pattern, not the environment in which the observer stayed. We instead wanted to see what would happen to pairs of equal targets in SLC patterns in which targets were adjacent only to their background and in which illumination affected the entire environment occupied by the observer, even under the Gelb lightning, in which stimuli may be strongly illuminated while the room still remained dark. Likewise, our results are not comparable with other studies aimed at replicating Jameson and Hurvich, the purpose of which was not necessarily to test lightness constancy per se. Flock and Noguchi (1970), for instance, specifically tested ‘brightness constancy’ as something different from lightness constancy: Their aim was to test the hypothesis of a brightness darkening effect, or a ‘–’ function as Flock and Noguchi (1970) dubbed it, related to the darkest target.

## Conclusions and Questions for the Future

Our findings contradicted both Jameson and Hurvich (1961) and those who failed to replicate their results, in the following ways: (a) We found that, in very general terms, illumination affected mostly the target on the black background, regardless of the illumination level. This indicated a target/background ratio dependent effect of illumination on lightness, that is, the effect of illumination on equal luminance targets was driven by target-to-background contrast polarity. Such finding was neither in line with Jameson and Hurvich’s results, nor with illumination-independent constancy findings. (b) We found that, in general, both targets in SLC configurations (except for the configuration *reduced*) appeared darker in the low illumination range, and the magnitude of SLC was reduced (Rudd & Rieke, 2016), though magnitudes were significantly different (over 1 Munsell step) only compared with the magnitudes of the double Gelb lighting condition for configurations *enhanced* and *ramps*. (c) However, with regard to configurations *enhanced* and *ramps*, the target on the white background did not get darker but remained virtually constant in lightness across the *normal* illumination range, as predicted by the anchoring theory (Economou et al., 2007). (d) We found that the effect of illumination interacted not only with the background but also with other surfaces not adjacent to the target, and such additional effects on lightness increased as the illumination on the configurations increased. This suggests that the effect of illumination intensity on a specific target was not driven by the absolute luminance of the target, nor was it only modulated by the target/background luminance ratio (Wallach’s ratio rule): Other photometric information within the configuration (e.g., other surfaces within the display) may modulate the lightness outcome in connection to the intensity of physical illumination.

The two-range illumination account remains, naturally, a hypothesis that requires further testing. In particular, it is important to verify what happens to the SLC illusion as illumination is further decreased and increased beyond the values we achieved and with both types of illumination (ambient and Gelb-like).

Here are a few questions that we find intriguing. With reference to scotopic vision: How dark can we go before the SLC illusion virtually disappears while the configuration is still visible? With reference to the two-range hypothesis: Will lightness evaluations and SLC magnitude stay constant if the intensity of the Gelb illumination is further increased? With reference to the Gelb lighting isolation hypothesis: Assuming the possibility of achieving very high luminance readings with our equipment for configurations illuminated by the light reflected by other surfaces (e.g., comparable with the 0 f/stop condition in Experiment 2), will the matching results still be consistent with those for the Gelb lighting that gives rise to the same luminance readings? With reference to the different types of SLC configurations employed: How is the lightness/brightness appearance of the other surfaces embedded in the modified SLC configurations (*reduced*, *enhanced*, and *ramps*) affected by variations in physical illumination? With reference to the experimental method: Will other types of lightness measurements (e.g., a lightness adjustment method) for the SLC illusion, applied to the illumination conditions we employed, deliver results similar to those we reported? With reference to lightness and illumination in general: How would other lightness/brightness illusions behave in experimental setups comparable with the ones we employed in Experiments 1, 2, and 3?

As those questions imply, we have possibly only scratched the surface of a new line of empirical investigations that could allow for deeper understanding of lightness perception as illumination varies.

## Note

1 It is however true that in lightness/brightness literature, simultaneous contrast configurations (Figure 1(a)) are often treated as brightness phenomena, in particular in the past century. Presumably this is because, without clear information about illumination (e.g., transversal illumination edges), some consider as impossible the distinction between those two perceptual dimensions, and such impossibility would lead to a brightness representation of the configuration (e.g., Blakeslee, Reetz, & McCourt, 2008). Other researchers, however, disagree with such view. For instance, Gilchrist et al. (1999) developed a theory that can account for simultaneous contrast without the need to refer to illumination as a mediated top-down construct; Rudd (2010; Experiment 2) showed that observers can estimate brightness by filtering out contrast induction, thus showing that observers can distinguish between lightness and brightness even in contrast displays; Zavagno et al. (2011) offered logical arguments against the notion of “brightness constancy” and its usefulness in perceptual terms.

## References

[bibr1-2041669518787212] Agostini T. Bruno N. (1996) Lightness contrast in CRT and paper-and-illuminant displays. Perception & Psychophysics 58: 250–258. 8838167 10.3758/bf03211878

[bibr2-2041669518787212] Agostini T. Galmonte A. (2002) A new effect of luminance gradient on achromatic simultaneous contrast. Psychonomic Bulletin and Review 9: 264–269. 12120788 10.3758/bf03196281

[bibr3-2041669518787212] Anderson B. Kim J. (2009) Image statistics do not explain the perception of gloss and lightness. Journal of Vision 9((10): 1–17. doi:10.1167/9.11.10. 10.1167/9.11.10 20053073

[bibr4-2041669518787212] Arend L. E. Spehar B. (1993) Lightness, brightness, and brightness contrast: 1. Illuminance variation. Perception & Psychophysics 54: 446–456. 8255707 10.3758/bf03211767

[bibr5-2041669518787212] Baddeley R. Attewell D. (2009) The relationship between language and the environment. Information theory shows why we have only three lightness terms. Psychological Science 20: 1100–1107. 19656339 10.1111/j.1467-9280.2009.02412.x

[bibr6-2041669518787212] Baddeley R. Attewell D. Patel S. (2010) Real world lightness constancy is bad (and why), but perfectly matched to language and memory. Perception 39 (ECVP Abstract Supplement): 8.

[bibr7-2041669518787212] Bergström S. S. (1994) Color constancy: Arguments for a vector model for the perception of illumination, color, and depth. In: Gilchrist A. (ed.) Lightness, brightness and transparency, Hillsdale, NJ: Lawrence Erlbaum Associates, pp. 257–286.

[bibr8-2041669518787212] Blakeslee B. McCourt M. E. (2012) When is spatial filtering enough? Investigation of brightness and lightness perception in stimuli containing a visible illumination component. Vision Research 60: 40–50. 22465541 10.1016/j.visres.2012.03.006 PMC3340531

[bibr9-2041669518787212] Blakeslee B. Reetz D. McCourt M. E. (2008) Coming to terms with lightness and brightness: Effects of stimulus configuration and instructions on brightness and lightness judgments. Journal of Vision 8: 3.1–3.14 doi:10.1167/8.11.3. 10.1167/8.11.3 PMC3176629 18831597

[bibr10-2041669518787212] Bressan P. (2006) The place of white in a world of grays: A double-anchoring theory of lightness perception. Psychological Review 113: 526–553. 16802880 10.1037/0033-295X.113.3.526

[bibr11-2041669518787212] Cataliotti J. Gilchrist A. (1995) Local and global processes in surface lightness perception. Perception & Psychophysics 57: 125–135. 7885811 10.3758/bf03206499

[bibr12-2041669518787212] Daneyko O. Zavagno D. (2008) Simultaneous lightness contrast with non-adjacent ramps and Gelb illumination. Perception 37 (ECVP Abstract Supplement): 104.

[bibr13-2041669518787212] Economou E. Zdravkovic S. Gilchrist A. (2007) Anchoring versus spatial filtering accounts of simultaneous lightness contrast. Journal of Vision 7((2): 1–15. Retrieved from http://journalofvision.org/7/12/2/, doi:10.1167/7.12.2. 10.1167/7.12.2 17997644

[bibr14-2041669518787212] Facchin, A., Zavagno, D., & Daini, R. (2017). The glare effect test and the impact of age on luminosity thresholds. *Frontiers in Psychology*, *8*: 1132. doi: 10.3389/fpsyg.2017.01132. 10.3389/fpsyg.2017.01132 PMC5492864 28713326

[bibr15-2041669518787212] Flock H. R. Noguchi K. (1970) An experimental test of Jameson and Hurvich’s theory of brightness contrast. Perception & Psychophysics 8: 129–136.

[bibr16-2041669518787212] Gelb A. (1929) Die Farbenkonstanz der Sehdinge [The color of seen things]. In: von Bethe A. (ed.) Handbuch der Normalen und Patologischen Physiologie Vol. 12, Berlin, Germany: Julius Springer, pp. 594–678.

[bibr17-2041669518787212] Gibson J. J. (1979) The ecological approach to visual perception, Orlando, FL: Houghton Mifflin.

[bibr18-2041669518787212] Gilchrist A. (1979) The perception of surface blacks and whites. Scientific American 240: 112–123. 451524 10.1038/scientificamerican0379-112

[bibr19-2041669518787212] Gilchrist A. (1994) Introduction: Absolute versus relative theories of lightness perception. In: Gilchrist A. (ed.) Lightness, brightness and transparency, Hillsdale, NJ: Lawrence Erlbaum Associates, pp. 1–34.

[bibr20-2041669518787212] Gilchrist A. (2006) Seeing black and white, New York, NY: Oxford University Press.

[bibr21-2041669518787212] Gilchrist A. (2016) On the ecological validity of computer displays. Perception 46: 767–771. 10.1177/0301006617699664 28622758

[bibr22-2041669518787212] Gilchrist A. Kossyfidis C. Agostini T. Li X. Bonato F. Cataliotti J. Economou E. (1999) An anchoring theory of lightness perception. Psychological Review 106: 795–834. 10560329 10.1037/0033-295x.106.4.795

[bibr23-2041669518787212] Haimson B. R. (1974) The response criterion, the stimulus configuration, and the relationship between brightness contrast and brightness constancy. Perception & Psychophysics 16: 347–354.

[bibr24-2041669518787212] Jacobsen A. Gilchrist A. (1988) The ratio principle holds over a million- to-one range of illumination. Perception & Psychophysics 43: 1–6. 3340493 10.3758/bf03208966

[bibr25-2041669518787212] Jameson D. Hurvich L. M. (1961) Complexities of perceived brightness. Science 133: 174–179. 13789338 10.1126/science.133.3447.174

[bibr27-2041669518787212] Kingdom A. A. (2011) Lightness, brightness and transparency: A quarter century of new ideas, captivating demonstrations and unrelenting controversy. Vision Research 51: 652–673. 20858514 10.1016/j.visres.2010.09.012

[bibr28-2041669518787212] Koffka K. (1935) Principles of Gestalt psychology, New York, NY: Harcourt, Brace & World.

[bibr29-2041669518787212] Logvinenko A. D. Adelson E. H. Ross D. A. Somers D. (2005) Straightness as a cue for luminance edge classification. Perception & Psychophysics 67: 120–128. 15912876 10.3758/bf03195016

[bibr30-2041669518787212] MacEvoy S. P. Paradiso M. A. (2001) Lightness constancy in primary visual cortex. PNAS 98: 8827–8831. doi:10.1073/pnas.161280398. 11447292 10.1073/pnas.161280398 PMC37520

[bibr31-2041669518787212] Motoyoshi I. Nishida S. Sharan L. Adelson E. H. (2007) Image statistics and the perception of surface qualities. Nature 447: 206–209. 17443193 10.1038/nature05724

[bibr32-2041669518787212] Noguchi K. Masuda N. (1971) Brightness changes in a complex field with changing illumination: A re-examination of Jameson and Hurvich’s study of brightness constancy. Japanese Psychological Research 13: 60–69.

[bibr33-2041669518787212] Packer O. Williams D. R. (2003) Light, the retinal image, and photoreceptors. In: Shevell S. K. (ed.) The science of color, 2nd ed Boston, MA: Elsevier, pp. 41–102.

[bibr34-2041669518787212] Ross W. D. Pessoa L. (2000) Lightness from contrast: A selective integration model. Perception and Psychophysics 62: 1160–1181. 11019614 10.3758/bf03212120

[bibr53-2041669518787212] Rudd, M. E. (2010). How attention and contrast gain control interact to regulate lightness contrast and assimilation: A computational neural model. *Journal of Vision*, *10*(14): 40, 1–37. doi:10.1167/10.14.40. 10.1167/10.14.40 21196510

[bibr35-2041669518787212] Rudd M. E. Rieke F. (2016) Brightness in human rod vision depends on slow neural adaptation to quantum statistics of light. Journal of Vision 16((23): 1–25. doi:10.1167/16.14.23. 10.1167/16.14.23 27903008

[bibr36-2041669518787212] Rudd M. E. Zemach I. K. (2007) Contrast polarity and edge integration in achromatic color perception. Journal of the Optical Society of America 24: 2134–2156. 17621319 10.1364/josaa.24.002134

[bibr37-2041669518787212] Saunders J. E. Jarvis J. R. Wathes C. M. (2008) Calculating luminous flux and lighting levels for domesticated mammals and birds. Animal 2: 921–932. 22443672 10.1017/S1751731108002012

[bibr38-2041669518787212] Schirillo J. A. Shevell S. K. (2002) Articulation: Brightness, apparent illumination, and contrast ratios. Perception 31: 161–169. 11922130 10.1068/p09sp

[bibr39-2041669518787212] Schmid A. C. Anderson B. L. (2014) Do surface reflectance properties and 3-D mesostructure influence the perception of lightness? Journal of Vision 14((24): 1–24. doi:10.1167/14.8.24. 10.1167/14.8.24 25074902

[bibr40-2041669518787212] Schubert F. E. (2006) Light-emitting diodes, New York, NY: Cambridge University Press.

[bibr41-2041669518787212] Taya R. (1990) Lightness perception in a complex field: A re-examination of the brightness contrast study of Jameson and Hurvich. Japanese Psychological Research 32: 1–19.

[bibr42-2041669518787212] Todorović D. (2006) Lightness, illumination, and gradients. Spatial Vision 19: 219–261. 16862841 10.1163/156856806776923407

[bibr43-2041669518787212] von Helmholtz H. (1866/1925) Helmholtz’s treatise on physiological optics, New York, NY: Optical Society of America.

[bibr44-2041669518787212] Wallach H. (1948) Brightness constancy and the nature of achromatic colors. Journal of Experimental Psychology 38: 315–336. 10.1037/h0053804 18865234

[bibr45-2041669518787212] Zavagno D. Caputo G. (2005) Glowing grays and surface white: The photo-geometric factors of luminosity perception. Perception 34: 261–274. 15895626 10.1068/p5095

[bibr46-2041669518787212] Zavagno D. Daneyko O. (2012) The effect of non-adjacent luminance gradients on simultaneous lightness contrast. In: Leth-Steensen C. Schoenherr R. (eds) Proceedings of the 28th Annual Meeting of the International Society for Psychophysics, Ottawa, Canada: International Society for Psychophysics, pp. 48–53.

[bibr47-2041669518787212] Zavagno D. Daneyko O. Actis-Grosso R. (2015) Mishaps, errors, and cognitive experiences: On the conceptualization of perceptual illusions. Frontiers in Human Neuroscience 9((190): 1–5. doi:10.3389/fnhum.2015.00190. 25918504 10.3389/fnhum.2015.00190 PMC4394699

[bibr48-2041669518787212] Zavagno D. Daneyko O. Agostini T. (2011) Measuring the meter: On the constancy of lightness scales seen against different backgrounds. Behavior Research Methods 43: 215–223. 21287112 10.3758/s13428-010-0035-y

[bibr49-2041669518787212] Zavagno D. Daneyko O. Sakurai K. (2011) What can pictorial artifacts teach us about light and lightness? Japanese Psychological Research 53: 448–462.

[bibr51-2041669518787212] Zele A. J. Cao D. (2015) Vision under mesopic and scotopic illumination. Frontiers in Psychology|Perception Science 5: 1594 doi:10.3389/fpsyg.2014.01594. 10.3389/fpsyg.2014.01594 PMC4302711 25657632

